# Classic Scrapie in Sheep with the ARR/ARR Prion Genotype in Germany and France

**DOI:** 10.3201/eid1308.070077

**Published:** 2007-08

**Authors:** Martin H. Groschup, Caroline Lacroux, Anne Buschmann, Gesine Lühken, Jacinthe Mathey, Martin Eiden, Séverine Lugan, Christine Hoffmann, Juan Carlos Espinosa, Thierry Baron, Juan Maria Torres, Georg Erhardt, Olivier Andreoletti

**Affiliations:** * Friedrich-Loeffler-Institut, Insel Riems, Germany; † Institut National de la Recherche Agronimique, Toulouse, France; ‡ Justus-Liebig–University Giessen, Giessen, Germany; § Centro de Investigación en Sanidad Animal, Madrid, Spain; ¶ Agence Française de Sécurité Sanitaire des Aliments, Lyon, France; 1 These authors contributed equally to this study.

**Keywords:** Prion, scrapie, bovine spongiform encephalopathy, research

## Abstract

In the past, natural scrapie and bovine spongiform encephalopathy (BSE) infections have essentially not been diagnosed in sheep homozygous for the A^136^R^154^R^171^ haplotype of the prion protein. This genotype was therefore assumed to confer resistance to BSE and classic scrapie under natural exposure conditions. Hence, to exclude prions from the human food chain, massive breeding efforts have been undertaken in the European Union to amplify this gene. We report the identification of 2 natural scrapie cases in ARR/ARR sheep that have biochemical and transmission characteristics similar to cases of classic scrapie, although the abnormally folded prion protein (PrP^Sc^) was associated with a lower proteinase-K resistance. PrP^Sc^ was clearly distinct from BSE prions passaged in sheep and from atypical scrapie prions. These findings strongly support the idea that scrapie prions are a mosaic of agents, which harbor different biologic properties, rather than a unique entity.

## Abstract 2

We report 2 natural scrapie cases in sheep carrying the ARR/ARR prion genotype, which is believed to confer resistance against classic scrapie and bovine spongiform encephalopathy.

Transmissible spongiform encephalopathies (TSEs) are fatal neurodegenerative diseases in sheep and goats (scrapie), cattle (bovine spongiform encephalopathy [BSE]), and humans (Creutzfeldt-Jakob disease [CJD]). A variant form of CJD (*1*) was discovered in 1996 and was linked to the BSE epidemic in the United Kingdom and elsewhere. Classic scrapie is caused by a variety of prion strains that can be distinguished from one another by their biologic and biochemical features (*2*). Recently, so-called atypical scrapie strains that have remarkably different biochemical and transmission characteristics have been discovered (*3*,*4*). Although the transmissibility of a particular sheep scrapie isolate to nonhuman primates has been demonstrated (*5*), no epidemiologic data have linked scrapie in small ruminants to human CJD cases (*6*). TSE susceptibility in sheep is controlled mainly by polymorphisms in the monocistronic *PRNP* gene that encodes for normal cellular protein (PrP^C^). Three major mutations are associated with sheep susceptibility or resistance to classic scrapie and BSE: at codons 136 (A or V), 154 (R or H), and 171 (R, Q, or H) (*7*). Animals with genotypes V^136^R^154^Q^171^/VRQ, ARQ/VRQ, ARQ/ARQ , and VRQ/ARH PrP are most susceptible to scrapie (*8*). In the past 20 years, no TSE cases have been found in ARR/ARR sheep in Europe, although thousands of scrapie-diseased animals have been genotyped. However, 1 report, albeit heavily questioned, has been made in the literature of a possible case in an ARR/ARR sheep in Japan (*9*). Therefore, this genotype was considered to confer full resistance to BSE and scrapie (*7*) (for a full review see [*10*]). To minimize the risk of humans acquiring TSE by consuming animal products, massive breeding programs involving PrP-genotyping of millions of sheep were initiated in the European Union (EU).

However, the successful transmission of BSE prions to ARR/ARR sheep showed that the resistance of this genotype toward the TSE agent was not absolute (*11*). Recently, the identification of previously unrecognized so-called atypical scrapie in sheep of various genotypes, including ARR/ARR, has reinforced this statement (*4*). We report here the identification and characterization of 2 natural classic scrapie cases in sheep of the ARR/ARR genotype, which are clearly different from BSE and atypical scrapie.

## Methods

### ELISA, Scrapie-associated Fibril, and Conventional Immunoblots

For ELISA detection of PrP^Sc^, commercial TSE rapid tests, TeSeE Sheep/Goat (Bio-Rad, Marnes-la-Coquette, France), were used according to the manufacturer’s recommendations. Scrapie-associated fibrils from the brain stem of infected ovines were purified and immunoblotted, according to the protocol by the World Organization for Animal Health (*12*). In this assay, we used the monoclonal antibody L42, which binds the 145–150 sequence of PrP (YEDRYY). Visualization was achieved by using the chemiluminescence substrate CDP-Star (Tropix, Bedford, MA, USA) and the Bio-Rad VersaDoc imaging system, and signals were analyized by using the Quantity One quantification software (Bio-Rad).

The TeSeE Western Blot Kit (Bio-Rad) was used according to the manufacturer’s recommendations. This kit uses the monoclonal antibody SHa-31, which binds the 145–152 sequence of PrP(YEDRYYRE).

### Proteinase K (PK) Resistance Assay

Ten percent of brain homogenates from the brain of a 5-year-old sheep with scrapie in France, designated S83, were analyzed by the TeSeE Sheep/Goat ELISA as recommended by the manufacturer. Each sample was diluted in PrP^Sc^-negative ARR/ARR sheep 10% brain homogenate until an optical density (OD) of 1.5 to 1.7 was obtained in the ELISA. Equilibrated homogenate aliquots were submitted to PK digestion with a concentration ranging from 50 to 500 µg/mg. PrP^Sc^ was precipitated (as in the basic TeSeE Sheep/Goat ELISA) and the pellet dissolved in 25 µL buffer C1, incubated for 5 min at 100°C, and diluted 12-fold in R6 reagent. Samples were run in triplicate and detected by using the TeSeE Sheep/Goat ELISA.

### Bioassay

Twenty microliters of 10% brain homogenates were intracerebrally inoculated into C57Bl6, RIII, Vm, and Tgshp XI mice that overexpress the ovine PrP^ARQ^, into Tg338 mice that overexpress the ovine PrP^VRQ^, and into Tgbov XV mice that overexpress the bovine PrP. Incubation times were recorded, and tissue samples from clinically affected mice were collected and preserved.

### Lesion Profiling and Paraffin-Embedded Tissue (PET) Blot

Lesion profiles were established by following the standard method of Fraser and Dickinson (*13*) and using 6 brains per isolate. For the PET blots, we used sections from positive transgenic mice (*14*).

### PRNP Sequencing

DNA was extracted from brain tissue of the scrapie-positive sheep with the QIAamp DNA Mini Kit (QIAGEN, Hilden, Germany). PCR-amplification and sequencing of a 970-bp PCR fragment, including the complete coding region of the *PRNP,* were done as described previously (*15*). Additionally, the PCR fragment was cloned with the pGEM-T Easy Vector System (Promega GmbH, Mannheim, Germany). Plasmid DNA of 12 recombinant clones was sequenced, and these sequences were compared with GenBank *PRNP* sequence U67922.

## Results

### Histories of 2 ARR Homozygous Sheep with Scrapie

The case in an ARR homozygous sheep in Germany was initially diagnosed during 2004 in the course of the intensified EU-wide testing of fallen stock and slaughter animals. A recent retrospective genotyping study on all 230 small ruminant TSE cases diagnosed in Germany between 2000 and 2006 led to the identification of this ARR/ARR case.

**Figure 1:**
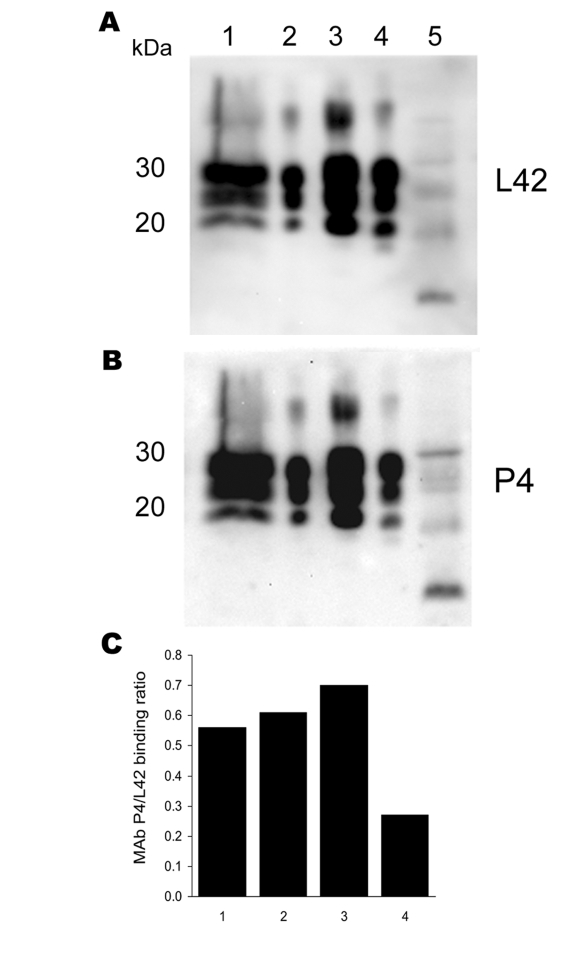
Antibody-binding patterns of the prion protein (PrP^Sc^) associated with cases of ARR/ARR scrapie in France and Germany. A) and B) Western blots showing the differences in monoclonal antibody (MAb) P4 binding compared with the internal standard MAb L42 of PrP^Sc^ derived from S115/04 (ARR/ARR Germany), S83 (ARR/ARR France), ovine ARQ/ARQ bovine spongiform encephalopathy (BSE), and S95 (classic scrapie) cases. Banding intensities were quantified by photoimaging, and binding ratios were calculated. Note the significantly weaker P4 binding to the ovine BSE sample. Lane 1, S115/04; lane 2, S83; lane 3, S95; lane 4, ovine BSE; lane 5, atypical S15. C) Relative MAb binding ratios for lane nos. 1–4 in the Western blots shown in A) and B).

This animal showed borderline reactivity in the Bio-Rad TeSeE rapid test on the brain stem (initial result, OD 0.279; cut-off 0.217; test repetition in duplicate OD 0.259/0.256; cut-off 0.219) and was positive in the Western blot confirmation test (Figure 1). The animal (S115/04) was a 2- to 4-year-old ewe of the black-headed German mutton breed. Apart from a general loss of condition, the animal did not show any clinical signs suggestive of scrapie. After the official confirmation of the case, all animals in the herd of origin (n = 1,297) were genotyped regarding PrP codons 136, 154, and 171. The frequencies of PrP genotypes in sheep >1 year of age (n = 871) were 46.7% (ARR/ARQ), 35.4% (ARR/ARR), 16.9% (ARQ/ARQ), 0.7% (ARR/VRQ), and 0.1% for each of the genotypes ARQ/VRQ, ARR/AHQ, and AHQ/ARQ. In accordance with the EU regulations, all sheep not carrying at least 1 ARR haplotype were slaughtered, and rapid tests for TSE were conducted. No further TSE case was detected among these sheep. The origin of the infection in this outbreak remains unknown, but rams originating from another flock in Germany with classic scrapie were apparently used in this flock in 1999.

We also examined brain, tonsil, or retropharyngeal lymph node samples from >1,700 sheep with clinically suspected scrapie, collected in France from 1993 through 2001, and identified a single case in a sheep with the ARR/ARR genotype. This animal, S83, was a 5-year-old sheep born in 1995 that had some clinical symptoms of scrapie but for which no detailed symptoms are described. The brain stem sample of S83 was positive by ELISA in the Bio-Rad TeSeE Sheep/Goat rapid test (OD 2.4; cut-off 0.212) and gave a clearly positive signal on Western blot (Figure 1).

Tonsil and retropharyngeal lymph nodes from this animal were negative for scrapie by ELISA and Western blot. In addition, conventional histologic examination found a severe *Listeria* infection, but no vacuoles, in the brain stem. No detailed information was available on the flock of origin and the genetic structure of that flock. No further scrapie cases were reported until 2001; the farm was then closed.

### Genetic Analysis

In addition to the routine genotyping at codons 136–154 and 171, *PRNP* gene sequences from the 2 scrapie cases were determined by sequencing of PCR amplificates from brain-derived DNA to compensate for the possibility of a genetic chimerism in blood. For both scrapie cases S115/04 and S83, *PRNP* sequence analysis unanimously showed the homozygous *PRNP* genotype ARR/ARR. There were no differences between the *PRNP* sequences determined by direct sequencing of the PCR products and by sequencing of 12 cloned PCR fragments for each case, which excludes the possibility that *PRNP* haplotypes other than ARR were present in the analyzed tissue.

### Biochemical Properties of PrP^Sc^

**Figure 2:**
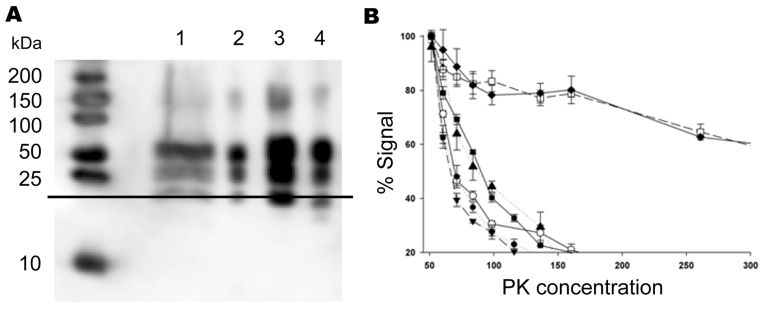
Biochemical characterization of the prion protein (PrP^Sc^) associated with ARR/ARR cases in France and Germany. A) Western blot (stained by monoclonal antibody L42) illustrating that protein kinase (PK)– treated ovine bovine spongiform encephalopathy (BSE) PrP^Sc^ has ≈1-kDa lower molecular mass than PrP^Sc^ from the scrapie cases. Lane 1, S115/04, molecular mass (MM) 20.95 kDa; lane 2, S83, MM 19.96 kDa; lane 3, S95 classic scrapie, MM 19.64 kDa; lane 4, ovine BSE, MM 18.85 kDa. B) PrP^Sc^ PK sensitivity measured by using brain from S83 scrapie case (▲), ARR/ARR BSE in sheep (○), ARQ/ARQ BSE in sheep (●), BSE from bovines (■), an ARR/ARR atypical scrapie case (▼), and 20 randomly selected isolates from sheep with scrapie in France (2 cases shown, represented as □ and ◆). PrP^Sc^ ELISA measurements were performed by using the TeSeE Sheep/Goat rapid test (Bio-Rad) after brain homogenate digestion using a PK concentration ranging from 50 µg/mL to 500 µg/mL. Three tests were performed for each sample and PK concentration.

The Western blot of S115/04 and S83 showed the 3-banded PrP^Sc^ pattern that is typically associated with scrapie. The molecular masses of the nonglycosylated PrP^Sc^ bands were ≈21 kDa, and both animals lacked the 12-kDa PrP^Sc^ band seen in atypical scrapie (Figure 1). Additionally, the nonglycosylated PK-treated PrP^Sc^ bands of S115/04 and S83 showed lower electrophoretic mobility than PrP^Sc^ derived from BSE-infected sheep (ARQ/ARQ). The monoclonal antibody P4, which recognizes PrP^Sc^ derived from scrapie-affected sheep, but has a lower affinity to BSE PrP^Sc^, clearly detected both S115/04- and S83-derived PrP^Sc^ and, to a substantially lesser extent, PrP^Sc^ from a BSE-infected sheep. These results strongly support the contention that both isolates differ from those that cause BSE and atypical scrapie (Figure 1; Figure 2, panel A).

The limited amount of sample material available from case S115/04 prevented any further biochemical characterization. For case S83, however, it was possible to compare the PK resistance of PrP^Sc^ with that of 20 randomly selected classic scrapie cases, 1 case of BSE in an ARR/ARR sheep, and 1 atypical case in an ARR/ARR sheep. S83 PrP^Sc^ was found to have a significantly lower level of PK resistance than that found in classic scrapie PrP^Sc^. However, resistance was similar to that of PrP^Sc^ in experimentally BSE-infected ARR/ARR sheep and PrP^Sc^ levels of animals with atypical scrapie (Figure 2, panel B).

### Bioassay in Transgenic Mice

Samples from both infected sheep were inoculated into a panel of conventional (C57Bl6, RIII, and VM) mice, transgenic bovinized (Tgbov XV) mice, and ovinized (ARQ Tgshp XI [unpub. data], VRQ Tg 338) mice. These transmission experiments are ongoing. However, for the isolate S83 from France, results in transgenic ovinized VRQ mice (Tg338) are available.

In intracerebrally inoculated Tg338 mice (20 µL of a 10% brain homogenate), nervous symptoms consistent with TSE developed after a mean incubation period of 309 ± 35 days. In all mice, PrP^Sc^ was detected by using Western blot. For Tg 338 mice, the mean incubation period was 239 ± 32 days for the atypical scrapie isolate, and the incubation time for BSE prion previously passaged through ARQ/ARQ sheep was 534 ± 25 days.

**Figure 3:**
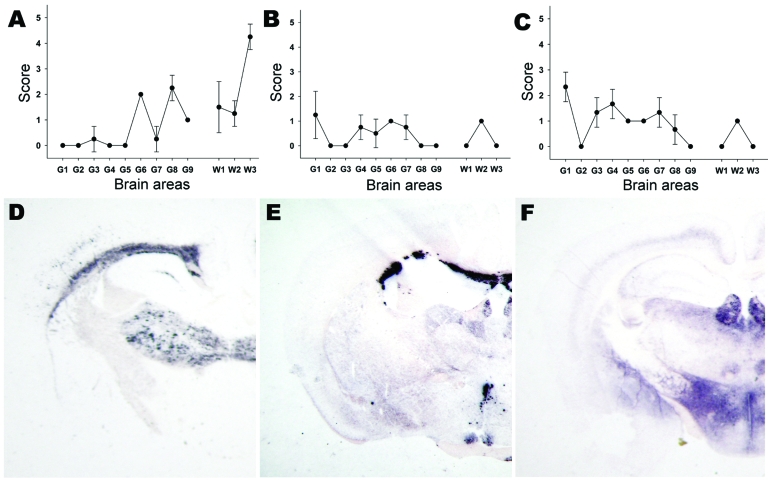
Lesion profiling (A, B, C) and paraffin-embedded tissue blot characterization of prion protein (PrP^Sc^) deposition at thalamic level (D, E, F). Tests were performed by using formalin-fixed brain from Tg338 mice (expressing the VRQ PrP ovine variant) inoculated with (A, D) ARR/ARR atypical case (B, E) bovine spongiform encephalopathy (BSE) brain from an ARR/ARR sheep (intracerebral inoculation), and (C, F) case S83. Each lesion profile was carried out by using 6 animals. Detection of PrP^Sc^ was achieved by using the monoclonal antibody Sha31.

Both lesion profile and PET blot PrP^Sc^ distribution in Tg 338 mice enabled a clear differentiation between the S83 isolate, atypical scrapie, and BSE in sheep (Figure 3). The lesion profile observed in Tg338 (Figure 3, panel A) inoculated with atypical scrapie was similar to that described in published data for 11 atypical scrapie cases in France (*16*).

**Figure 4:**
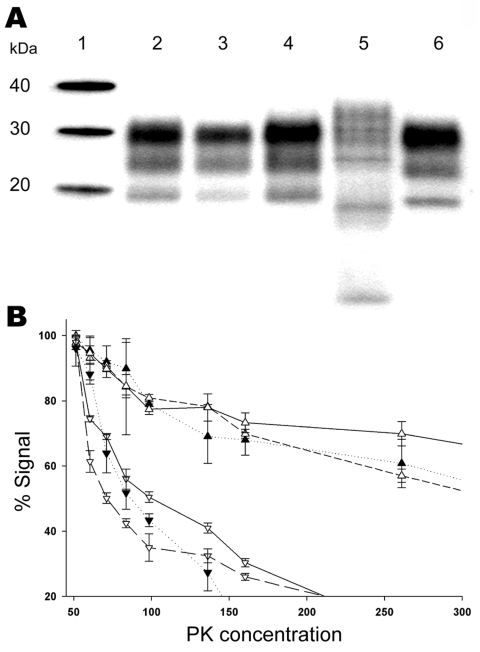
Biochemical properties of prion protein (PrP^Sc^) associated with the ARR/ARR scrapie case S83 from France after passage in Tg 338 VRQ mice. A) Western blot mobility of the original S83 ARR/ARR case (lane 3) and S83 passaged in Tg338 (lane 4) were similar and comparable to a classic scrapie isolate (Langlade, lane 2). PrP^Sc^ WB profile of ARR/ARR bovine spongiform encephalopathy (BSE) in sheep (lane 6) and profiles of atypical scrapie case isolates (lane 5) passaged into Tg338 mice were readily distinguishable by their banding pattern or electromobility. B) PrP^sc^ protein kinase (PK) sensitivity of the original S83 isolate (▼) and a classic scrapie isolate (Langlade) (△) compared with S83 (▽) and Langlade (▲) that had been passaged in Tg338 (2 different mice for each isolate). Triplicate ELISA measurements were performed by using the TeSeE Sheep/Goat rapid test (Bio-Rad), after brain homogenate digestion with PK concentration ranging from 50 µg/mL to 500 µg/mL.

S83-isolate–infected Tg338 mice PrP^Sc^ reproduced a Western blot signature similar to that of the initial cases (electromobility and glycotype) and was distinct from that of PrP^Sc^ purified from Tg338 mice infected with either BSE in sheep or atypical scrapie (Figure 4, panel A). After passage of the 1) S83 isolate, 2) isolates from the atypical scrapie case and BSE in sheep, and 3) 3 independent classic scrapie isolates passaged in Tg338 mice, the PK resistance of the PrP^Sc^ was measured (Figure 4, panel B). Because Tg338 mice overexpress the VRQ sheep allele and, consequently, PrP^Sc^ produced in this mouse model derives from the conversion of VRQ-PrP^C^, the finding that PrP^Sc^ PK resistance in these mice was similar to that of the original isolate was surprising. Thus, the lower PrP^Sc^ PK resistance observed in S83 isolate seems not to depend on the *PRNP* sheep genotype but is rather an intrinsic property of the scrapie strain involved.

## Discussion

Epidemiologic and genetic data collected in recent decades have indicated that sheep carrying the PrP^C^-encoding ARR haplotype only are resistant to natural TSE infections, whereas those carrying the homozygous VRQ or ARQ haplotypes are highly susceptible. This assumption has been supported by genotyping data for thousands of sheep with scrapie worldwide: only 1 homozygous ARR carrier has been found. A case of classic scrapie in an ARR/ARR sheep in Japan was reported more than a decade ago (*7*). However, the validity of this diagnosis has been heavily challenged when it could not be reconfirmed independently, because no suitable frozen or formalin-fixed material was available. Our current report shows that classic scrapie cases can occur in homozygous ARR sheep and suggests that even the former report may have been an imperfect first demonstration of such a case.

The apparent resistance of sheep of the ARR/ARR genotype to both natural scrapie and experimental BSE has triggered national authorities since the late 1990s (e.g., United Kingdom, the Netherlands, France), and since 2001 the EU, to implement a genetic selection and culling policy in sheep to protect the human food chain from small ruminant TSEs and to eradicate TSE from affected flocks.

This global approach, even if valuable, considered scrapie as a single entity and did not take into account any kind of TSE agent biodiversity. Sheep TSE agents have strikingly different abilities to replicate in hosts expressing a spectrum of PrP variants. Sheep with genotype ARQ/ARQ in scrapie flocks are commonly affected by the disease (*8*). However, a historical sheep scrapie brain pool (SSBP-1) transmits easily to VRQ homozygous or heterozygous sheep but not to ARQ/ARQ animals (*17*). Similarly atypical scrapie cases (including the “Nor98” type) are more frequently found in AHQ and AF_141_RQ haplotype carriers than in sheep carrying exclusively other haplotypes. However, atypical cases have also been found in ARR/ARR animals (*18*,*19*). Similarly, the ability of BSE to develop in ARR/ARR sheep was observed after experimental parenteral inoculation. However, in this case, higher transmission rates and shorter incubation periods were observed in sheep of the other genotypes, such as ARQ/ARQ, and AHQ/AHQ (*20*,*21*). The susceptibility of ARR/ARR sheep to an oral BSE challenge was reported most recently (*22*).

Both BSE and atypical scrapie PrP^Sc^ have a characteristic molecular signature, which allows a rapid and reliable biochemical discrimination from each other and from classic scrapie PrP^Sc^ (*3*,*23*). In both cases of scrapie in ARR/ARR sheep in France and Germany, abnormal PrP^Sc^ harbored features (apparent molecular mass and glycotype) that were similar to those observed in classic scrapie. However, at least in the S83 case, PrP^Sc^ seemed to have a remarkably lower PK resistance than that observed in a panel of scrapie isolates. This observation sustains the idea that the involved agent could belong to a particular scrapie agent group that cannot be directly identified by using the current biochemical criteria for TSE agent discrimination.

The successful propagation of the S83 isolate in Tg338 mice that express the ovine VRQ haplotype and the persistence of its original biochemical signature (including the low PK resistance) allow the inference that this scrapie agent could also be present and could naturally propagate in sheep that harbor genotypes other than ARR/ARR. The transmissibility and contagiousness of the S83 isolate are currently under investigation in experimentally challenged sheep. These experiments should produce a better understanding of the susceptibility of each genotype to this agent and its capacity to spread efficiently in sheep flocks.

The discovery of these 2 cases clearly indicates that the genetic resistance of ARR/ARR sheep to the so-called classic scrapie agent is not absolute. It also provides evidence that, rather than being a single entity, scrapie is a mosaic of infectious agents harboring different biologic properties in its natural host. Finally, although many thousands of cases of classic scrapie have been reported in sheep of other PrP genotypes and hundreds of thousands of rapid tests have been performed in Europe since the implementation of active TSE surveillance in small ruminants began in 2001, the discovery of these 2 ARR/ARR cases supports the idea that such infections are extremely rare.

## References

[1] Ironside JW, Sutherland K, Bell JE, McCardle L, Barrie C, Estebeiro K, A new variant of Creutzfeldt-Jakob disease: neuropathological and clinical features. Cold Spring Harb Symp Quant Biol. 1996;61:523–30. 9246478

[2] Bruce ME. TSE strain variation. Br Med Bull. 2003;66:99–108. 10.1093/bmb/66.1.99 14522852

[3] Benestad SL, Sarradin P, Thu B, Schonheit J, Tranulis MA, Bratberg B. Cases of scrapie with unusual features in Norway and designation of a new type, Nor98. Vet Rec. 2003;153:202–8. 12956297 10.1136/vr.153.7.202

[4] Buschmann A, Biacabe AG, Ziegler U, Bencsik A, Madec JY, Erhardt G, Atypical scrapie cases in Germany and France are identified by discrepant reaction patterns in BSE rapid tests. J Virol Methods. 2004;117:27–36. 10.1016/j.jviromet.2003.11.017 15019257

[5] Baker HF, Ridley RM, Wells GA. Experimental transmission of BSE and scrapie to the common marmoset. Vet Rec. 1993;132:403–6. 8488658 10.1136/vr.132.16.403

[6] van Duijn CM, Delasnerie-Laupretre N, Masullo C, Zerr I, de Silva R, Wientjens DP, Case-control study of risk factors of Creutzfeldt-Jakob disease in Europe during 1993–95. European Union (EU) Collaborative Study Group of Creutzfeldt-Jakob disease (CJD). Lancet. 1998;351:1081–5. 10.1016/S0140-6736(97)09468-3 9660576

[7] Hunter N. Prion protein (prnp) genotypes and natural scrapie in closed flocks of Cheviot and Suffolk sheep in Britain. In: Court L, Dodet B, editors. Transmissible subacute spongiform encephalopathies: prion diseases, Paris: Elsevier; 1996. p. 47–50.

[8] Baylis M, Goldmann W, Houston F, Cairns D, Chong A, Ross A, Scrapie epidemic in a fully PrP-genotyped sheep flock. J Gen Virol. 2002;83:2907–14. 12388827 10.1099/0022-1317-83-11-2907

[9] Ikeda T, Horiuchi M, Ishiguro N, Muramatsu Y, Kai-Uwe GD, Shinagawa M. Amino acid polymorphisms of PrP with reference to onset of scrapie in Suffolk and Corriedale sheep in Japan. J Gen Virol. 1995;76:2577–81. 10.1099/0022-1317-76-10-2577 7595361

[10] Opinion of the Scientific Panel on Biological Hazards on. the breeding programme for TSE resistance in sheep. The EFSA Journal. 2006;382:1–46.

[11] Houston F, Goldmann W, Chong A, Jeffrey M, Gonzalez L, Foster J, Prion diseases: BSE in sheep bred for resistance to infection. Nature. 2003;423:498. 10.1038/423498a 12774113

[12] World Organization for Animal Health (OIE). Manual of diagnostic tests and vaccines for terrestrial animals (mammals, birds and bees). Paris. Organization. 2004;642–53.

[13] Fraser H, Dickinson AG. The sequential development of the brain lesion of scrapie in three strains of mice. J Comp Pathol. 1968;78:301–11. 10.1016/0021-9975(68)90006-6 4970192

[14] Andreoletti O, Simon S, Lacroux C, Morel N, Tabouret G, Chabert A, PrPSc accumulation in myocytes from sheep incubating natural scrapie. Nat Med. 2004;10:591–3. 10.1038/nm1055 15156203

[15] Luhken G, Buschmann A, Brandt H, Eiden M, Groschup MH, Erhardt G. Epidemiological and genetical differences between classical and atypical scrapie cases. Vet Res. 2007;38:65–80. 10.1051/vetres:2006046 17156738

[16] Le Dur A, Beringue V, Andreoletti O, Reine F, Lai TL, Baron T, A newly identified type of scrapie agent can naturally infect sheep with resistant PrP genotypes. Proc Natl Acad Sci U S A. 2005;102:16031–6. 10.1073/pnas.0502296102 16239348 PMC1276041

[17] Goldmann W, Hunter N, Smith G, Foster J, Hope J. PrP genotype and agent effects in scrapie: change in allelic interaction with different isolates of agent in sheep, a natural host of scrapie. J Gen Virol. 1994;75:989–95. 10.1099/0022-1317-75-5-989 7909834

[18] Buschmann A, Luhken G, Schultz J, Erhardt G, Groschup MH. Neuronal accumulation of abnormal prion protein in sheep carrying a scrapie-resistant genotype (PrPARR/ARR). J Gen Virol. 2004;85:2727–33. 10.1099/vir.0.79997-0 15302966

[19] Moum T, Olsaker I, Hopp P, Moldal T, Valheim M, Moum T, Polymorphisms at codons 141 and 154 in the ovine prion protein gene are associated with scrapie Nor98 cases. J Gen Virol. 2005;86:231–5. 10.1099/vir.0.80437-0 15604451

[20] Foster JD, Parnham D, Chong A, Goldmann W, Hunter N. Clinical signs, histopathology and genetics of experimental transmission of BSE and natural scrapie to sheep and goats. Vet Rec. 2001;148:165–71. 11258721 10.1136/vr.148.6.165

[21] Ronzon F, Bencsik A, Lezmi S, Vulin J, Kodjo A, Baron T. BSE inoculation to prion diseases-resistant sheep reveals tricky silent carriers. Biochem Biophys Res Commun. 2006;350:872–7. 10.1016/j.bbrc.2006.09.137 17049491

[22] Andreoletti O, Morel N, Lacroux C, Rouillon V, Barc C, Tabouret G, Bovine spongiform encephalopathy agent in spleen from an ARR/ARR orally exposed sheep. J Gen Virol. 2006;87:1043–6. 10.1099/vir.0.81318-0 16528056

[23] Thuring CM, Erkens JH, Jacobs JG, Bossers A, van Keulen LJ, Garssen GJ, Discrimination between scrapie and bovine spongiform encephalopathy in sheep by molecular size, immunoreactivity, and glycoprofile of prion protein. J Clin Microbiol. 2004;42:972–80. 10.1128/JCM.42.3.972-980.2004 15004040 PMC356877

